# Automated spatio-temporal analysis of dendritic spines and related protein dynamics

**DOI:** 10.1371/journal.pone.0182958

**Published:** 2017-08-21

**Authors:** Vincent On, Atena Zahedi, Iryna M. Ethell, Bir Bhanu

**Affiliations:** 1 Department of Electrical and Computer Engineering, University of California Riverside, Riverside, CA, United States of America; 2 Department of Bioengineering, University of California Riverside, Riverside, CA, United States of America; 3 Department of Biomedical Sciences, School of Medicine, University of California Riverside, Riverside, CA, United States of America; Augusta University, UNITED STATES

## Abstract

Cofilin and other Actin-regulating proteins are essential in regulating the shape of dendritic spines, which are sites of neuronal communications in the brain, and their malfunctions are implicated in neurodegeneration related to aging. The analysis of cofilin motility in dendritic spines using fluorescence video-microscopy may allow for the discovery of its effects on synaptic functions. To date, the flow of cofilin has not been analyzed by automatic means. This paper presents Dendrite Protein Analysis (DendritePA), a novel automated pattern recognition software to analyze protein trafficking in neurons. Using spatiotemporal information present in multichannel fluorescence videos, the DendritePA generates a temporal maximum intensity projection that enhances the signal-to-noise ratio of important biological structures, segments and tracks dendritic spines, estimates the density of proteins in spines, and analyzes the flux of proteins through the dendrite/spine boundary. The motion of a dendritic spine is used to generate spine energy images, which are used to automatically classify the shape of common dendritic spines such as stubby, mushroom, or thin. By tracking dendritic spines over time and using their intensity profiles, the system can analyze the flux patterns of cofilin and other fluorescently stained proteins. The cofilin flux patterns are found to correlate with the dynamic changes in dendritic spine shapes. Our results also have shown that the activation of cofilin using genetic manipulations leads to immature spines while its inhibition results in an increase in mature spines.

## Introduction

**Fig 1:**
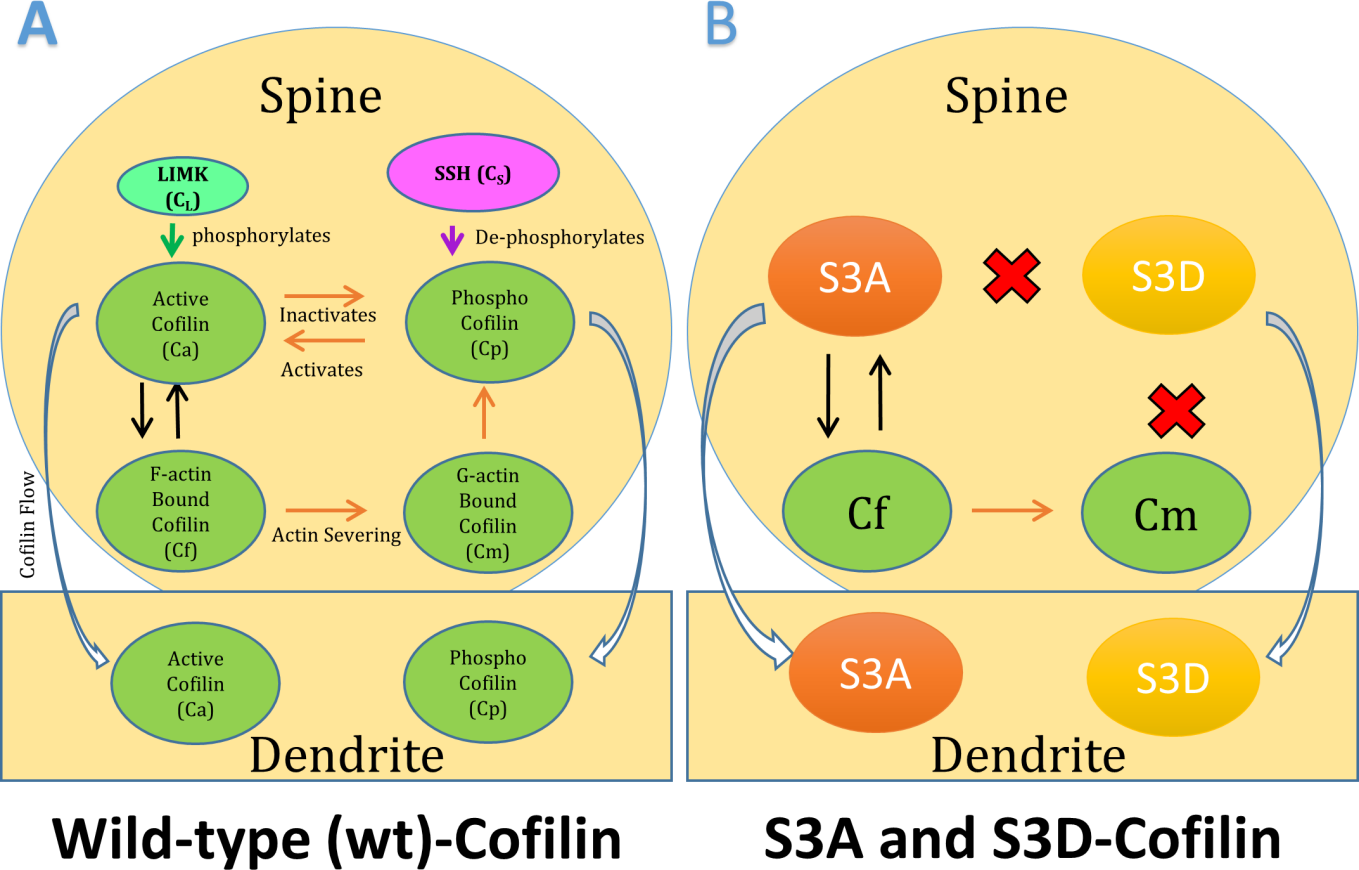
Cofilin dynamics in dendritic spines. A) When wild-type cofilin is active (Active Cofilin Ca) it binds to F-actin (F-actin Bound Cofilin Cf) and severs it into G-actin (G-actin Bound Cofilin Cm). When cofilin is phosphorylated (Phospho Cofilin Cp) on Ser3, it cannot bind the actin. This phospho-regulation is mediated by two upstream players, LIMK which phosphorylates cofilin, and SSH which dephosphorylates cofilin. Both Active and Phospho Cofilin can move from the spines into the dendrites. B) Cofilin-S3A, which cannot be phosphorylated, can bind to actin (Cf) and sever it into G-actin (Cm). However, Cofilin-S3D cannot bind to actin. S3A and S3D are not able to convert between each other, since they are different mutants. However, both may flow in and out of dendritic spine regions.

Dendritic spines are small protrusions located on the surface of neurons, which receive inputs from other neurons and are the active sites for neuronal communications called synapses. These synapses are often remodeled by the rapid turnover of the actin cytoskeleton, which is regulated by various actin-binding proteins [1, 2]. Cofilin is an actin-severing protein and its activity is regulated by phosphorylation at Ser3 [3, 4]. Cofilin mediated remodeling of the actin cytoskeleton is critical in regulating the shape and functionality of dendritic spines. Therefore, the localization and phosphorylation state of cofilin within dendritic spines can affect the synaptic functions. Cofilin-S3A is a constitutively active mutant form of cofilin, where the Ser3 is substituted to alanine, which can constitutively bind and remodel actin filaments, whereas in inactive form of Cofilin-S3D the serine is substituted with aspartate [1, 2]. The S3A mutant cannot be inactivated by phosphorylation, hence it is always in its active “severing” state, which leads to filamentous actin (F-actin) depolymerization, whereas the S3D substitution prevents the cofilin binding to F-actin, which reduces F-actin depolymerization. These two mutants are often used to study the mechanism of cofilin phospho-regulation in neurons [5, 6] and are shown in Fig 1. Although various studies have reported on the functional down-stream effects of the cofilin mutants, the changes in their dynamics have been relatively unexplored to date.

In this paper, we investigate the dynamics of the actin-severing protein, cofilin, and its effects on remodeling of dendritic spines. Dendritic spines contain the post-synaptic sites of excitatory synapses in the central nervous system (CNS) [7–11]. Dysregulation of dendritic spines can have a strong impact on brain functions and underlie cognitive decline associated with neurological diseases. Cofilin can regulate the remodeling of dendritic spines through the disassembly and reorganization of F-actin cytoskeleton, which provides the structure to dendritic spines. Elevated levels of cofilin have previously been shown to contribute to loss of synapses and spines in neurodegenerative disorders, such as Alzheimer’s disease (AD) [12, 13]. However, the precise mechanism underlying cofilin-mediated loss of synapses is unclear. Therefore, it is important to quantify the motility of cofilin and examine how the localization of cofilin affects dendritic spine shape.

Most previous studies involving the effects of proteins on neurons have primarily used manual examination, segmentation, and classification [14]. Most of these biological studies have used popular user-operated software such as ImageJ [15] to manually segment dendritic spines, other studies have used visualization systems such as Imaris or Neurolucida [16]. However, both Imaris and Neurolucida require z-stack information and are sensitive to parameter selection. These manual methods are prone to human bias and are extremely tedious and time-consuming processes when performed on multiple images. Because of this, it is advantageous to develop an image analysis software such as DendritePA to automatically segment dendritic spines and extract features for the analysis of live fluorescence videos.

To automatically relate cofilin motility with dendritic spine shapes, dendritic spines must be automatically segmented. Because of the small size of dendritic spines, it is very hard to acquire images with sufficient resolution and contrast to properly analyze the dynamic entities and structures. To compensate, many experiments use the maximum intensity projection of a z-stack instead of data from a single image. For our work, capturing these z-stacks would be disadvantageous, as we are examining two separate fluorescence channels; a green channel, which detects wild-type (wt)-Cofilin-GFP to assess cofilin motility and a red channel to detect tdTomato providing spine structural information. While it may be possible that overexpression of proteins cause artificial effects, our previous studies with the cofilin mutants have showed no adverse effects of GFP-tagged cofilin or TdTomato on neurons [5]. In addition, the genetic cofilin mutants cofilin-S3A and cofilin-S3D were used to study the effects of cofilin activity on its dynamics and spine shape. To analyze the spatiotemporal relationship between dendritic spines and cofilin, a time series of sufficient temporal resolution must be captured. Here we present a method that uses the spatiotemporal information of the video to improve the signal-to-noise ratio of each frame without having to acquire z-stack data.

In this paper, we present DendritePA, a novel automated pattern recognition system that analyzes protein localization in neurons using multi-channel florescence microscopy and relates it to dendritic spine shape and protein activity state. Unlike previous work, our DendritePA uses video bioinformatics algorithms to automatically obtain spatiotemporal pattern information on protein dynamics. The system is used specifically to examine the effects of cofilin motility on the shape and evolution of dendritic spines. Fluorescence microscopy is used because the pixel intensity is assumed to be proportional to the amount of stained proteins in the region. Due to the small size of cofilin, which is at the subpixel level or smaller than a pixel at 40x magnification, individual molecules of cofilin cannot be tracked. However, our DendritePA can estimate the changes in cofilin density within the dendritic spines by measuring their intensity levels. DendritePA also uses a spine energy representation derived from an existing motion pattern representation called gait energy image (GEI) to summarize spine motion into a single image. Doing so allows for the extraction of useful features that can be used to classify segmented spine shapes. By relating the spine shapes with the observed cofilin trafficking dynamics, it is possible to examine the underlying biological processes.

## Related work

Some preliminary work reported in this paper were originally presented at the International Conference on Pattern Recognition 2016 [17]. To the best of our knowledge, before our previous conference paper, cofilin has never been automatically quantified. Another actin-regulating protein paxillin, has previously been automatically analyzed in non-neuronal florescence images [18]. However, only paxillin dense regions that are clearly visible are examined. These paxillin dense regions are also sparse and appear much brighter than the rest of the cell allowing for simple segmentation. However, this is not the case for cofilin located in neurons as it is more uniformly distributed. Also, cofilin dense regions are not static, forming and dispersing over time, making tracking these clusters challenging. Bosch et al. [6] have manually studied the effect of cofilin localization on the remodeling of dendritic spines. They classified cofilin transport in dendritic spines into four patterns: persistent increase in concentration, transient increase, transient decrease, and persistent decrease. In their work, they found that cofilin transport patterns correlated with the remodeling of dendritic spine shapes.

There are some current methods that automatically inspect the flow of proteins in cells. Many of these techniques evaluate individual particle trajectories over time by using frame by frame object detection [19] and associating the objects across time. An issue with these techniques is that they do not perform well with high particle density and background noise. Another method is to separate the cell into regions and measure particle flux by the intensity level or protein density in the regions [20, 21]. Pecot et al. [21] designed an approach that involves partitioning the cell into predefined sections of set sizes and shape. By checking the quantity of particles or tracking changes in the intensity levels, they could estimate the flux of these particles through the boundaries between sections. A drawback of this approach is that regions must be rigid and the choice of region size affects the performance. Experiments on live samples utilized micro-fabricated patterns [22] to constrain the cell shape so that the partitioned regions remained consistent throughout the experiment.

To efficiently correlate cofilin motility with dendritic spine shape, spines must be automatically segmented and classified. Spine segmentation methods can be separated into two groups, classification-based [23] and centerline extraction based [24–28]. The classification-based methods classify individual pixels into various groups such as spine, dendrite, or background [23]. The software NeuronStudio by Rodriguez et al. [23] utilizes the pixel distance to the nearest surface point as a feature in classification, however, this can generate spurious spine detection and it is sensitive to noise. NeuronStudio also requires manual input by the user before the segmentation process can begin. Centerline extraction based methods involve detecting the backbone or central region of dendrites and segmenting spines by their relationship to the central region. Traditional methods may experience issues when the dendrite width varies along its orientation. In our previous work, the method [29] was used to detect a center region using gradient vector flow [30] instead of a thin backbone. However, this method does not completely capture the segmentation of a dendrite. Instead, in this paper, we choose to build upon the method used by Basu et al. [28] called 2dSpAn. This method uses a set of convolution kernels at varying angles to accurately segment a dendrite. This method can compensate for varying dendritic spine widths. However, the software requires user input of seed points for the kernels, does not consider dendrites with large curvatures, and may overestimate the size of the dendrite at the base of large spines. In our work, we address each of these issues.

Using segmented spine information, automated classification of shape type is critical in analyzing biological conditions. Basu et al. [28] utilized a decision tree method to classify spines by using neck length, spine height, and head width. An issue with these features arises when the resolution is low because they are measured in only a few pixels. This prompts an increased likelihood of measurement error and resulting in misclassifications. The primary feature utilized by DendritePA is an adaption of gait energy image (GEI) [31]. GEI is a spatiotemporal gait representation that has been widely used to characterize human walking patterns and has previously been shown to be highly effective for recognition of different individuals. DendritePA uses a spine energy image (SEI) along with other features in the classification of dendritic spine shapes such as mushroom, thin, and stubby. Unlike previous spine classification methods, SEI allows for the use of spatiotemporal information in classification.

As compared to previous work, the key contributions of our work are: a) Present DendritePA (protein analysis) software which is an automated, unbiased program that can be used to segment and track dendrite spines, analyze cofilin patterns in fluorescence live videos, and classify dendrite spine shape. b) Develop for the first time an automated algorithm suite to quantify the movement of cofilin in dendrites and spines, and correlate it to spine shape using multi-channel fluorescence live videos. c) Use spatiotemporal information to enhance the signal-to-noise ratio in videos and perform automated analysis of multiple fluorescent probes in time-lapse videos for tracking the local distribution of cofilin while simultaneously analyzing the effects on spine shape. d) Segment spines and dendrites using convolution kernels that can adapt to changing angles automatically. e) Automatically classify individual dendritic spine shapes using SEI and other features. f) Examine the effect of cofilin activation using wild-type cofilin, cofilin-S3A, and cofilin-S3D. Understanding the dynamics of cofilin motility and activation within sub-neuronal compartments is critical to understanding its function in regulating the morphological structure and functionality of synapses.

## Materials and methods

### Animal protocol

All animal care protocols and procedures were approved by the UC Riverside Animal Care & Use Program, which is accredited by AAALAC International, and animal welfare assurance number A3439-01 is on file with the Office of Laboratory Animal Welfare (OLAW).

#### Mice

Mice were obtained from Jackson laboratories, housed in an AAALAC-accredited facility under 12-h light/dark cycles and fed standard mouse chow. Food and water were provided *ad libitum*. All procedures were approved by the Institutional Animal Care and Use Committee at the University of California, Riverside.

#### Hippocampal neuron cultures

Cultures of hippocampal neurons were prepared from embryonic day 15 (E15) or E16 pups. Briefly, hippocampal cells were treated with papain (0.5 mg/ml) and DNase (0.6 μg/ml) for 20 min at 37°C, mechanically dissociated, and then plated on glass coverslips that had been pre-coated with poly-DL ornithine (0.5 mg/ml in borate buffer) and laminin (5 μg/ml in PBS). The hippocampal cells were cultured in Neurobasal medium with 25 μM glutamine, 1% penicillin-streptomycin, and B27 supplement (Invitrogen, Carlsbad, CA), under 5% CO_2_/10% O_2_ atmosphere at 37°C. Hippocampal neurons were transfected with ptdTomato and pcDNA3-EGFP-cofilin, pcDNA3-EGFP-cofilinS3A or pcDNA3- EGFP-cofilinS3D plasmids to express tdTomato and wt-Cofilin-GFP, cofilin-S3A-GFP or cofilin-S3D-GFP at 10 days in vitro (DIV) using a calcium phosphate method, as previously described [5].

#### Live imaging

Time-lapse imaging was performed on 14 DIV hippocampal cultures under an inverted fluorescent microscope (model TE2000; Nikon) with a 40x air Fluor objective and monitored by a 12-bit CCD camera (model ORCA-AG; Hamamatsu) using Image-Pro software (Media Cybernetics). During imaging, the cultures were maintained in Hank’s solution supplemented with 1.8 mM CaCl2, 0.45% glucose, and 0.1% BSA at 37°C and 5% CO2, and images were captured at 3 min intervals for 1 h. Cofilin was visualized by GFP fluorescence and dendritic spines were identified with tdTomato. Briefly, samples were encoded for blind analysis. In each experiment, 2–3 coverslips were analyzed for each condition. At least ten spiny pyramidal neurons were randomly imaged in each group.

### Development and use of DendritePA software

**Fig 2:**
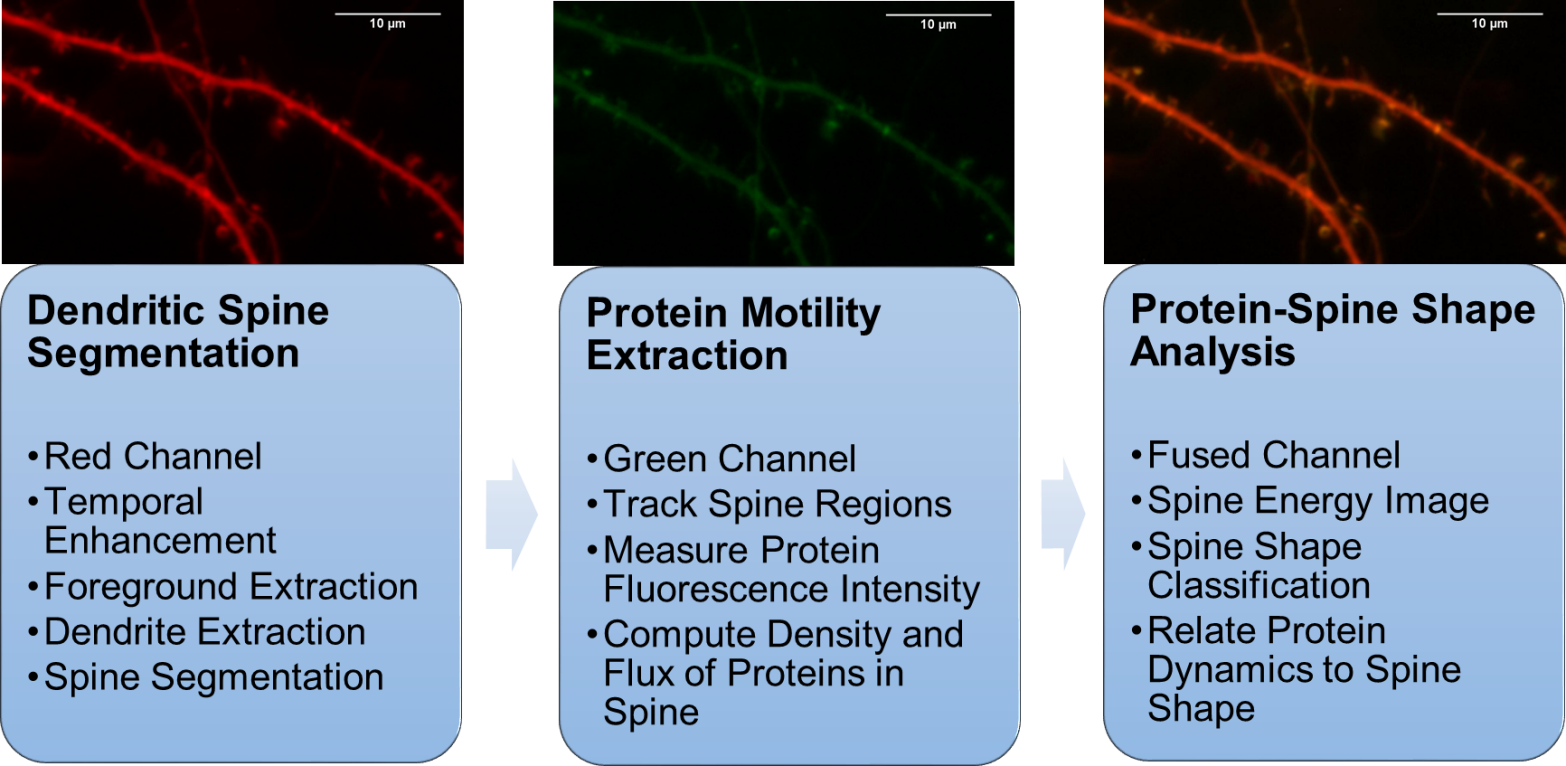
System overview diagram. DendritePA is designed with three subsystems: Dendritic spine segmentation, Protein Motility Extraction, and Protein-Spine Shape Analysis. Dendritic spine segmentation subsystem uses the red fluorescence channel to extract the foreground, central region, and spines. Protein motility extraction subsystem uses green fluorescence channel to measure cofilin levels and transport in spines. Protein-spine shape analysis subsystem uses both channels and temporal information to relate spine shape with cofilin flow.

The DendritePA is designed in a modular manner with three parts: Dendritic spine segmentation, protein motility extraction, and cofilin-spine shape analysis. A diagram of our workflow is shown in Fig 2. DendritePA was written and developed with MATLAB 2016a programming environment. The MATLAB source code, a stand-alone executable version of this algorithm, and supplied test data are available online at http://vislab.ucr.edu/SOFTWARE/software.php. DendritePA.m is the main program of the code and requires the following MATLAB toolboxes: Statistics and Machine Learning, Bioinformatics, Computer Vision System, Image Processing, Mapping, and System Identification. The standalone executable requires the installation of the 64-bit version of MATLAB Runtime R2016a (9.0.1) available at http://www.mathworks.com/products/compiler/mcr/.

### Dendritic spine segmentation

Dendritic spine segmentation is performed on the red color channel of our florescence data. This channel is stained with TdTomato, which fills the cell, providing structural information used for examining the cell morphology. Common issues with fluorescence microscopy include hazy background noise, lack of contrast, and bleaching of intensity over time [32]. The background may auto-fluoresce and structures such as other dendrites and axons that are out of focus may affect the structures of interest. To account for these issues, it is useful to preprocess a video. Top-hat filtering has been used to reduce background fluorescence [33]. For this work, a top-hat filter using a disk with a radius of 50 pixels was used on each frame. After completing top-hat filtering, a 3 by 3 median filter was also used to reduce noise. Previous methods have used histogram matching to correct for photo-bleaching. This step is important for segmentation of dendritic spines in later frames as well as getting the correct intensity of fluoresced proteins. Every frame after the first was histogram matched using the first frame as a reference [34].

#### Temporal maximum intensity projection

Dendritic spine segmentation begins by estimating the foreground in each frame. The foreground in our case is any pixel brightly illuminated by fluorescent proteins in a dendritic structure. DendritePA starts by computing the maximum intensity projection using all frames in a video. For a video consisting of *N*_*v*_ frames, this temporal maximum intensity projection (TMIP) is defined as follows:

$$T(x,y)=\underset{t}{\max}I(x,y,t),$$ (1)

where *I(x*,*y*,*t)* is the image or frame at time *t*. Since TMIP uses the maximum intensity of a pixel along the time dimension, a TMIP pixel will have a larger intensity if a structure strongly fluoresced at that location for any time in the video. The pixel value will be minimal for any background structures such as dendrites or axons outside of the focal distance. The TMIP is then max-min normalized producing filter whose values will be used as weights for enhancing the signal-to-noise ratio in each frame. For every frame, the TMIP is multiplied to the image as weights. This image enhance procedure is summarized in the following equation:

$$I'(x,y,t)=\frac{T(x,y)-T_{\min}}{T_{\max}-T_{\min}}*I(x,y,t).$$ (2)

**Fig 3:**
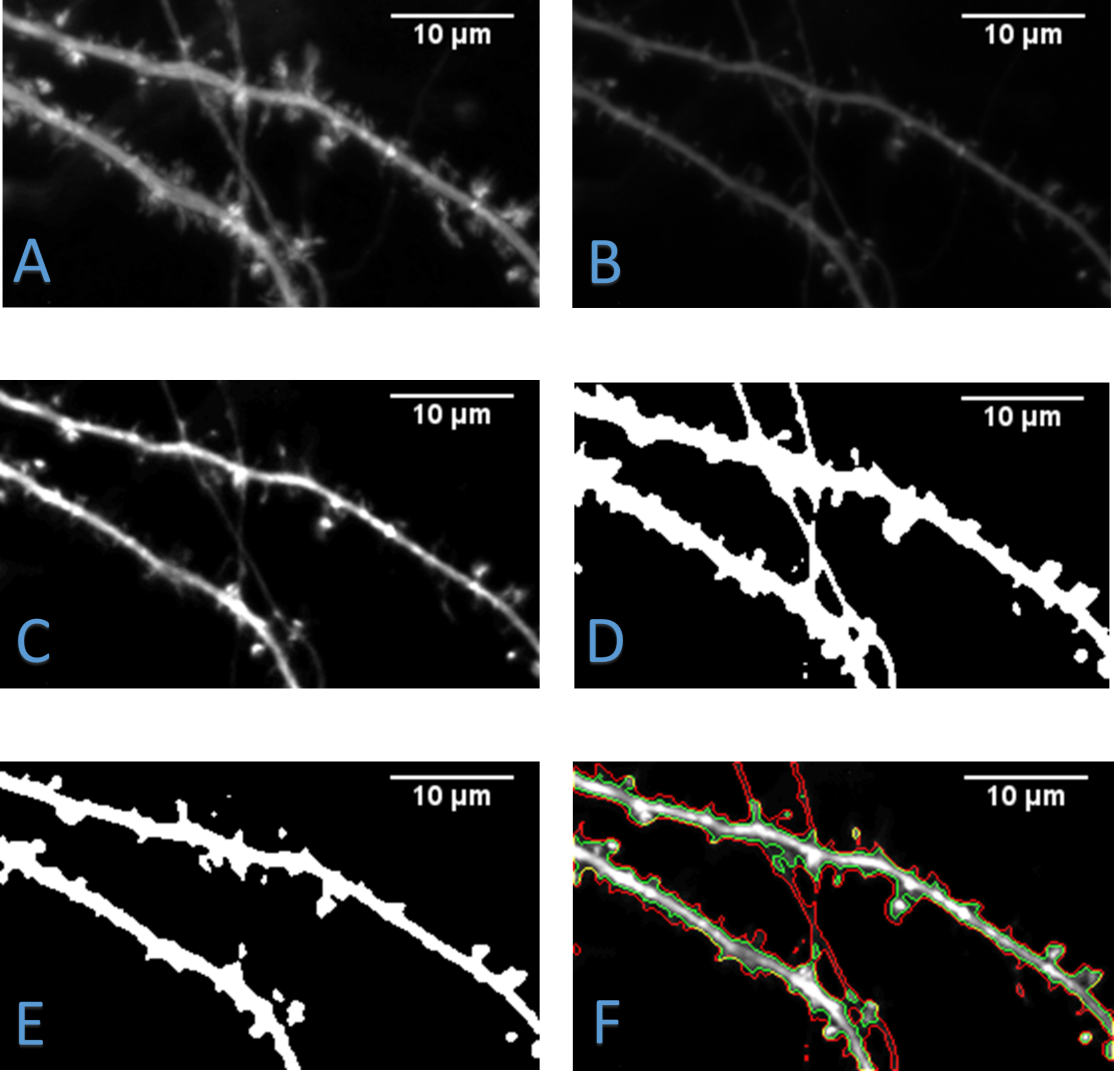
Effect of preprocessing video frames. A) Temporal maximum intensity projection (TMIP) computed from an image sequence of dendrites. B) An original frame from the image sequence. C) An enhanced image generated by combining the original frame with the TMIP. D) Extracted foreground segmentation done on the original frame without TMIP. E) Foreground extracted after enhancement with TMIP. F) An overlay of foreground contours with (green outline) and without (red outline) TMIP on enhanced frame. This reveals that TMIP significantly improves segmentation of foreground.

By preprocessing with a TMIP, structures that are brighter in the TMIP will be enhanced in each frame, while background structures such as axons or dendrites outside the depth of focus will be suppressed. Since the data in the present frame is utilized, no artifacts will be generated from the bright areas in the TMIP. The TMIP, an original image, and a temporally enhanced image are shown in Fig 3. TMIP is only applied for the segmentation step and not utilized in the cofilin analysis step. This is because it is undesirable to modify the intensity in such a way that would change the relative intensity differences between pixels. An initial segmentation of the dendrites and spines can now be computed by using the Otsu’s method [35]. This initial segmentation is the foreground which can be used to extract the central region of the dendrite. With the foreground computed, the contours are then acquired by removing all interior pixels of the foreground leaving only the outline.

#### Foreground and dendrite segmentation

**Fig 4:**
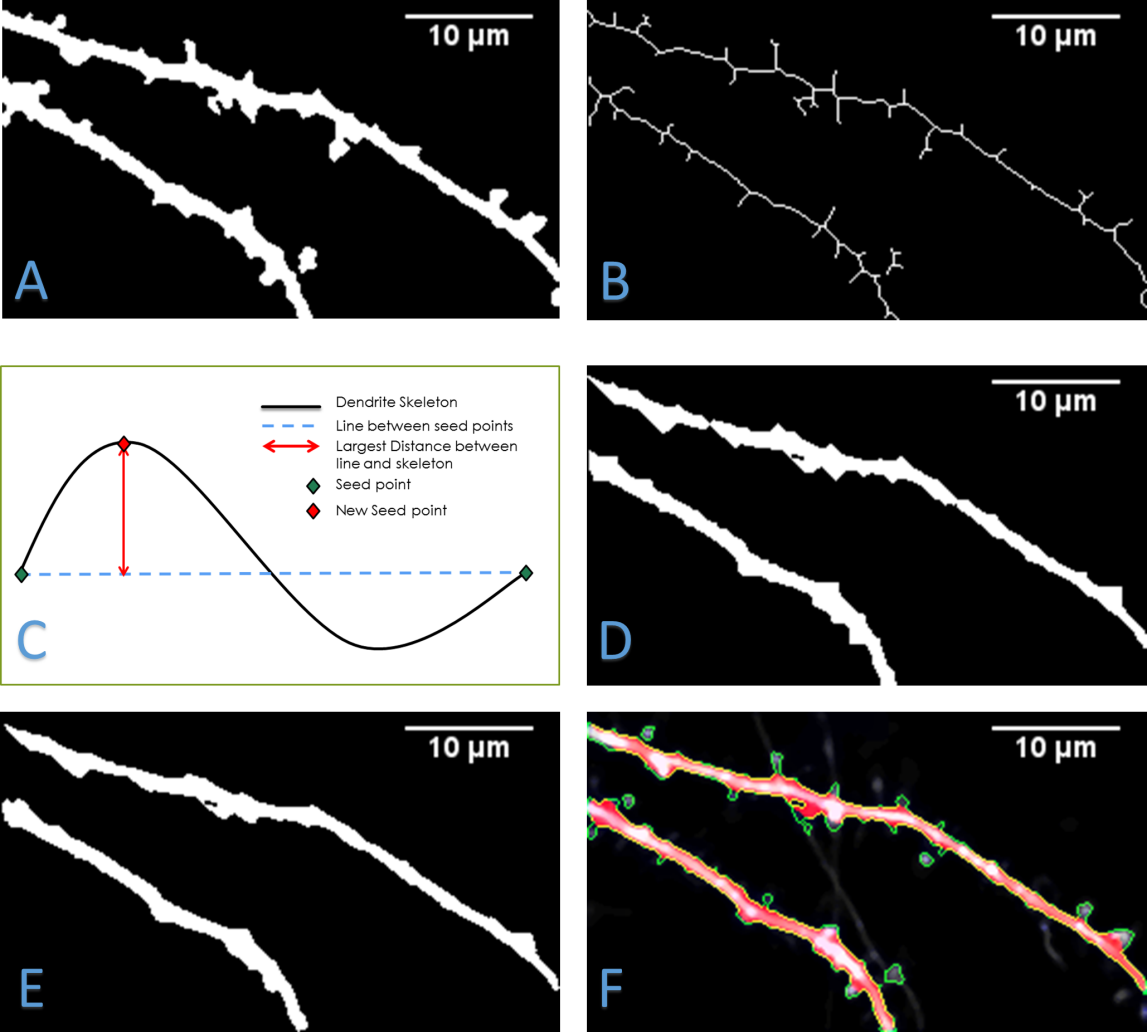
Dendrite segmentation. A) Foreground segmentation of one image. B) Skeletonization of the segmentation done by removing pixels from the peripheries until only an individual pixel remains. C) Diagram of piecewise linear approximation method used to automate dendrite segmentation. D) Dendrite segmentation using convolution kernels. E) Low pass filter output performed after the convolution step. F) Segmented foreground contours (green outline) and final dendrite segmentation (red) overlaid on to the original frame.

Upon computing the foreground, we segment the central regions or backbone of dendrites. Past strategies have relied on basic skeletonization of the initial segmentation until only a thin backbone remain. The skeleton of a foreground image (Fig 4A) is shown in Fig 4B. However, this skeleton does not give the best representation of a dendrite and does not give data regarding the changing width of the dendrite. Precise dendrite information is critical because numerous spine segmentation algorithms are dependent on accurately segmented dendrites. In our previous work [17], we used the method outlined in [29] to find the central region. This method used a modified gradient vector flow (GVF), in which vectors are orientated towards the center of a structure. Starting from every edge pixel, the algorithm follows the path of vectors until it encounters a vector greater than 90 degrees from the current vector. Both these pixels will now be marked as a central dendrite pixel. While this method is an improvement over skeletonization, it does not fully capture the outline of a dendritic spine as it will not detect the outer edges of a dendrite. Another issue is that the algorithm will not detect central regions near large dendrites or crossing structures. This is because the GVF vectors area oriented in a spiral for these regions and will not be 90 degrees from each other, which causes an infinite loop as it traces the vectors.

For this work, we adapted the convolution kernel method of [28] called 2dSpAn. In this method, a foreground segmentation containing spines and dendrites is computed either with manual thresholding or automatic Otsu’s method [35]. The user specifies two points on the dendrite. The angle of flow is computed based on the two points selected. Based on this angle, a set of two 3 by 3 convolution kernels is selected. Starting from one point, a convolution kernel is continuously applied until it reaches the other point. Next, starting from the opposite point, the complementary convolution kernel is applied until it reaches the first. The intersection of the two regions generated by the kernels is taken as the segmentation of the dendrite.

Because the kernels are chosen by the original angle of flow, this method fails for dendrites with large curvature. To automate this method and to account for large curvatures, we propose the following steps. The foreground segmentation is skeletonized and trimmed. The two endpoints of the dendrite skeleton that are furthest from each other are used as the first two seed points for the kernel method. Additional seed points can now be computed using piecewise linear approximation as shown in Fig 4C. A line connecting the two current seed points is computed and the dendrite skeleton pixel that is furthest from this line is evaluated. If the distance between the dendrite skeleton pixel and the closest point on the line is greater than distance *d* (which is set to 15 pixels for our experiments), then this skeleton pixel will be used a new seed point. The seed point generation step is repeated for all pairs of seed points until no more seed points can be generated. This allows the dendrite to be computed in a piecewise process, which accounts for large curvature in the dendrite. Once all seed points are found, the convolution kernel method of 2dSpAn is used to generate a dendrite segmentation as shown in Fig 4D.

Another issue with 2dSpAn is that for spines with large bases, a portion of the spine will be included in the dendrite segmentation. Also, by breaking the dendrite into piecewise segments, regions at the seed points will not be segmented perpendicular to the angle of flow. By applying a low pass filter to the dendrite segmentation contour, the method smooths contour of segmentation. This repairs both types of regions by reducing the high-frequency changes in the dendrite segmented contour as seen in Fig 4E. Fig 4F shows the contour of the foreground (green) and the segmentation of the dendrite (red) overlaid onto the original image.

#### Spine segmentation and declumping

**Fig 5:**
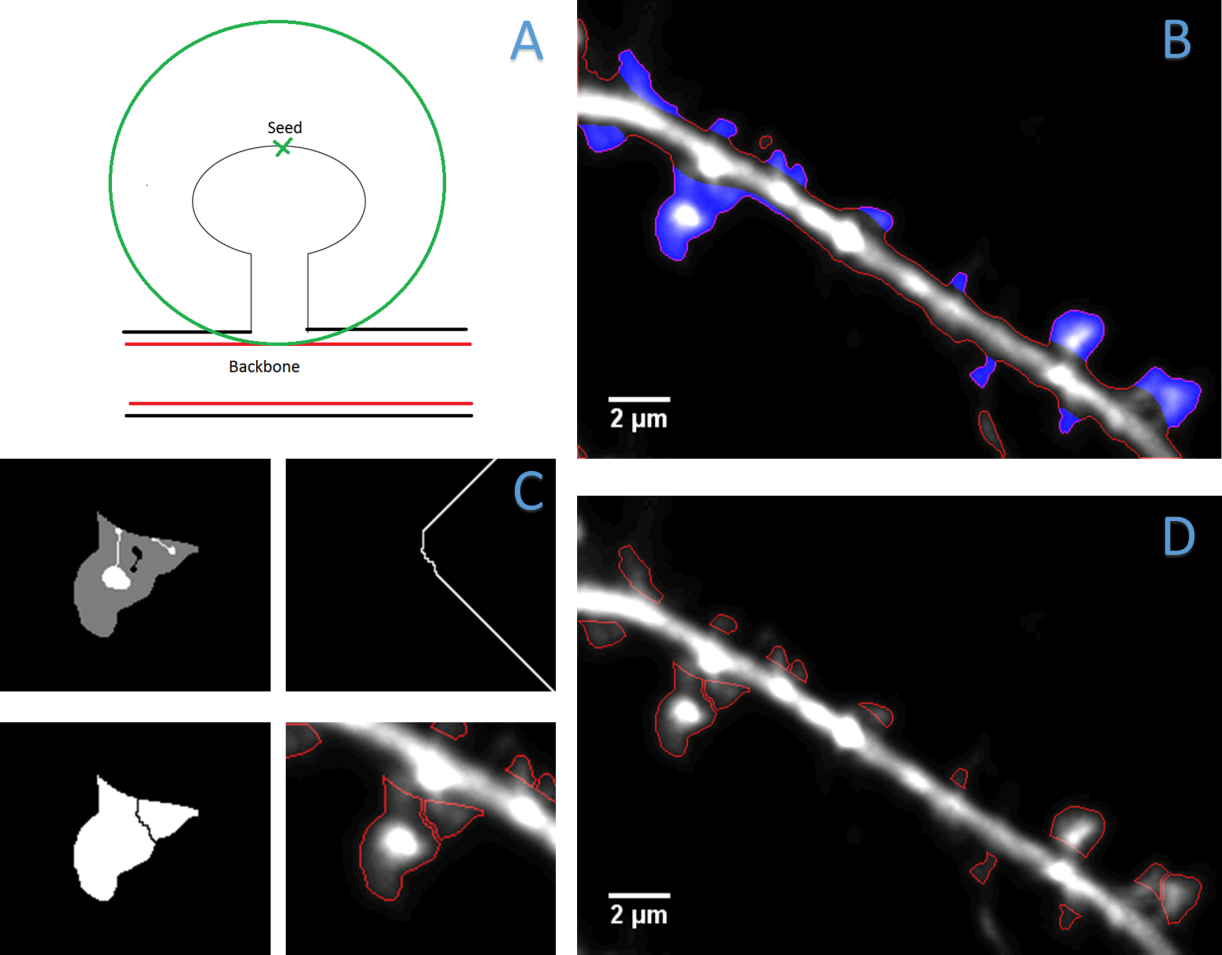
Spine segmentation. A) Diagram of dendritic spine contour extraction using a seed point. This contour is used as the initial segmentation of a dendritic spine. B) Spine segmentation (blue) and foreground contour (red) overlaid onto the image. C) Images of procedure to declump a segmented dendritic spine. Marker-controlled watershed composite image (top left), boundary generated by watershed algorithm (top right), declumped segmentation (bottom left), and contour of declumped segmentations overlaid onto image (bottom right). D) Final spine segmentation displaying split and declumped spines.

Once a segmentation of the dendrite is obtained, DendritePA begins the spine segmentation process. Using the generated backbone, a distance map is computed [36]. This distance map measures the distance of each foreground pixel to the closest dendrite pixel. Only pixels in the computed foreground contour are considered dendritic spine candidates. Using the inner distance map and the foreground contour, all regional maxima are used as possible dendritic spine detections. These regional maxima correlate with the furthest spine pixels from the central regions of the dendrite and are considered seed points for spine detection. All contour pixels that are closer to the seed point than the closest backbone pixel are considered a part of that spine as shown in Fig 5A. The end points of this contour are found and a line is drawn to connect them. This line represents the interface or boundary between the spine and its dendrite. The contour is now filled and will be used as the segmentation of the spine as displayed in Fig 5B.

While many spines will be properly segmented, some spines may be merged due to their close proximity to each other. Spines that are slightly touching at the base can be split by computing the distance to dendrite of every contour pixel. We trace these values and detect if a regional minimal distance is between two regional maximal distances. If the minimal distance is less than half of either maxima, draw a dividing line form the minima to closest dendrite pixel. For spines with severe overlap, we propose a marker control watershed method to declump the dendritic spines. For each spine region, examine the intensity and detect regional minima and maxima. If the centroid of two maxima are not parallel to the dendrite, connect the maxima with a line. This line must not cross any minimal regions. The same is done for any pair of regional minima as long as the line does not cross any maximal regions. Using the spine segmentation, connected maxima, and connected minima, a composite image is generated to serve as the markers for the watershed method. Background and regional minima are set to a low value of zero, regional maxima are set to a high value of 255 and the rest of the spine segmentation is set to a middle value. The composite image is inverted and the watershed will begin filling at the regional maxima. As the algorithm continues, the middle values will begin to form the watershed boundaries while taking the regional minima into account. The regional maxima were connected to reduce the number of watershed boundaries, while connected regional minima were used to aid in the shape of the boundary. The generated watershed boundary is applied to the spine mask, separating the segmentation in two. The declumping process is illustrated in Fig 5C and the final segmentations after splitting and declumping are shown in Fig 5D.

### Cofilin motility extraction

Cofilin motility extraction is performed exclusively on the green channel of our fluorescence microscopy videos. The channel shows GFP-labeled cofilin, (wt)-Cofilin-GFP, which allows for cofilin density to be visually analyzed. In order to analyze the motility of cofilin, at least two adjacent frames are needed. Because individual cofilin molecules exist at the subpixel level and cannot be resolved at our (40x) magnification, they cannot be tracked individually. Since the green intensity channel tracks the GFP-tagged cofilin proteins, the intensity is directly proportional to the amount of cofilin in the pixel. While the visual changes in cofilin density are difficult to examine by eye, the DendritePA is able to analyze this data by using spatiotemporal information in the red structural channel. Utilizing the previous dendritic spine segmentations, DendritePA can estimate the amount of cofilin contained in these structures. The framework starts by performing data association of segmented spines in neighboring frames to produce dendritic spine tracks. Association is performed by choosing the segmentation with the largest percentage of overlap with an existing track. The ratio of overlap is sufficient as the spines are attached to a fixed location on the dendrite. Most of the movement in dendritic spines is attributed to sway and shape change, whereas the sway of the dendrite itself is negligible. A new track may be created if the segmentation has little or no overlap with a track. Dendritic spines may not be detected in every frame as they may shrink into the dendrite or sway in and out of the z-axis, thereby going out of view. Because of this, a spine segmentation may be associated with any existing track if there is overlap with the last known location.

#### Cofilin flux

Since we are concerned with the flow of cofilin through sections of a cell, it is advantageous to relate this concept to fluid dynamics. The differential form of the continuity equation in fluid mechanics is written as:

$$\frac{\delta \rho }{\delta t}+div(f)=s,\;\mathrm{such}\;\mathrm{that}\;f=\rho v,$$ (3)

where *ρ* is the density of the fluid particles, *f* is the flux of the fluid through a boundary, *v* is the velocity, and *s* is a source term. To find the change in the amount of cofilin in the spine, we want to solve for the *div(f)* which is the “flux density” and represents the amount of flux entering or leaving a point. In florescence microscopy, fluorescence intensity levels are proportional to the amount of tagged proteins in a region. This leads to the follow proportionality formula:

$$\rho \propto \rho_{i}(t)=\sum_{\left(x,y)\in S(t\right)}i(x,y,t),$$ (4)

where *p*_*i*_*(t)* is the integrated density in spine *S(t)* at time *t*, and *i(x*,*y*,*t)* is the intensity of pixel *(x*,*y)*. Using integrated density accounts for changing spine area and is commonly used for analyzing fluorescence microscopy images [37]. It can be assumed that cofilin neither produced nor consumed in the spine. This allows the source term *s* to be set to zero for all calculations of flux. Also as there is only one boundary between the spine and the dendrite, cofilin flux must be either in or out of this boundary. Solving for *div(f)*, the continuity equation becomes:

$$div(f)\propto -\frac{\delta \rho_{i}(t)}{\delta t}.$$ (5)

Cofilin flux and density can now be compared by examining the intensity levels in the green cofilin channel. An increase in the intensity in a spine represents an increase in the cofilin density at the spine and a flux of cofilin into the spine. Conversely, a decrease in the intensity represents a decrease in cofilin density as well as a flux of cofilin out of the spine.

### Cofilin-spine shape analysis

After estimating the motility of cofilin and dendritic spines morphology, we correlate their effect on one another. The initial step is to automatically classify the shape of the dendritic spine using machine learning. To do this, we obtain the spine energy image representation of a spine. Generating the SEI starts by cropping the binary segmentation of each spine in a track. All binary spines images are then transformed so that the spine-dendrite boundary is aligned with the x-axis. These aligned cropped images are resized into 10x10 images. Given the registered binary images *B*_*t*_*(x*,*y)* at time *t* for a spine track of *N* frames, the spine energy image can be computed as follows:

$$S(x,y)=\frac{1}{N}\sum_{t=1}^{N}B_{t}(x,y).$$ (6)

**Fig 6:**
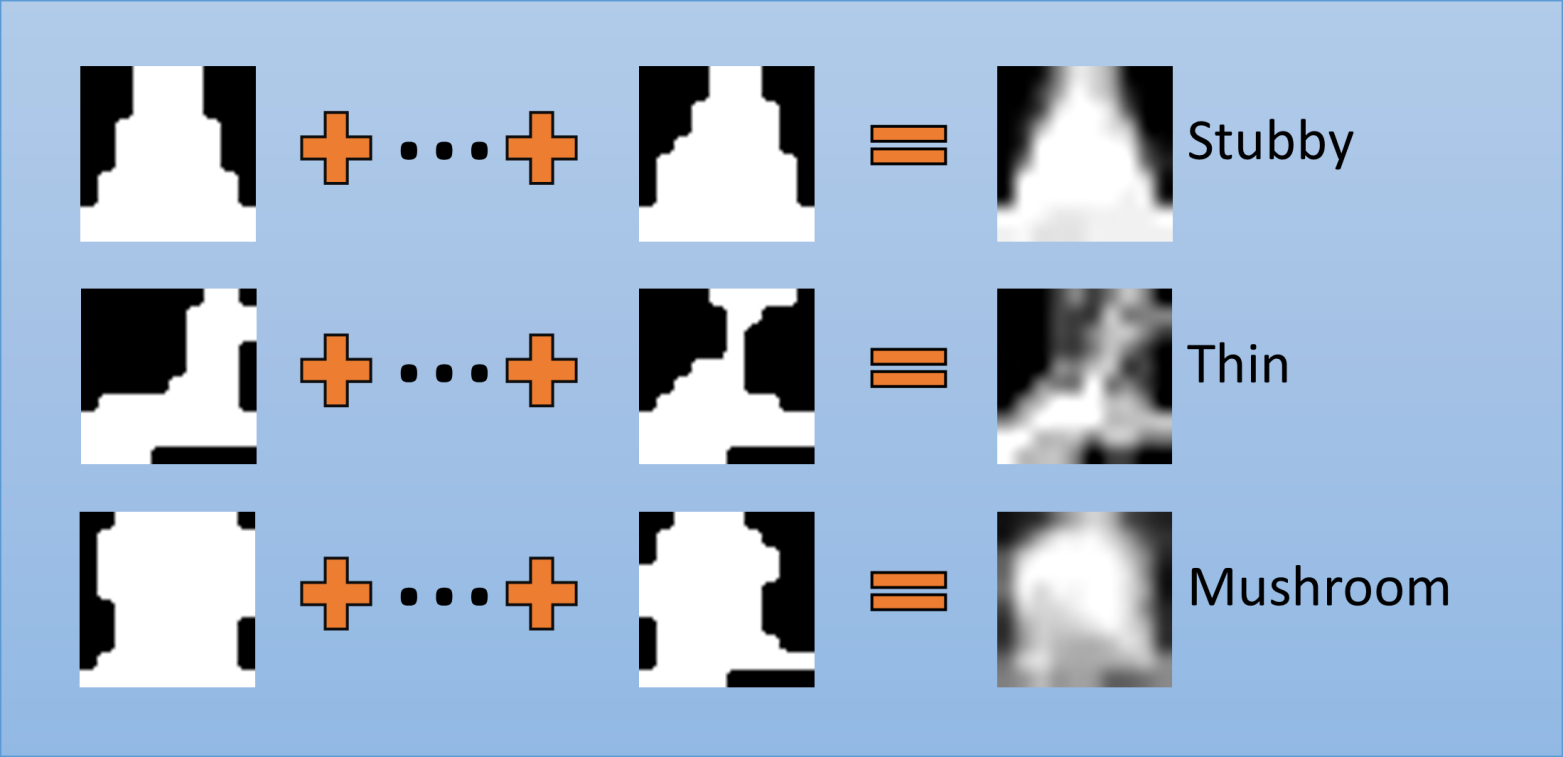
Spine energy image. Examples of aligned binary images and spine energy image for each class stubby, thin, and mushroom shaped dendritic spines. The aligned and resized spine segmentations of a single spine track are combined to produce a single spine energy image (images in the furthest right column). This image is a representation of the motion of the spine for the image sequence.

An example of the aligned binary images and SEI are shown in Fig 6. Since the SEI images are 10x10 pixels, the dimension of the feature vector is 100, which leads to a problem with the curse of dimensionality. This feature vector needs to be reduced while minimizing the loss of information. Recent studies [38, 39] have shown that local binary pattern applied to Gait Energy Image can reduce dimensionality. By applying uniform LBP [40] to SEI, the feature vector is reduced to 59 dimensions.

While SEI has been shown in our previous work [17] to classify spine tracks well, it cannot classify individual spine segmentations by itself. To classify individual spine, additional features must be used. Before being passed to a classifier the SEI feature vector is combined with the area, height, width, and average intensity of each spine. These features were used to train three classifiers: (1) discriminant analysis (DA) classifier, (2) k-nearest neighbor (KNN), and (3) error-correcting output codes (ECOC) multiclass model. The discriminant analysis classifier trains by fitting a Gaussian distribution to each class. New data is compared to each class distribution and assigned to the class with the lowest misclassification cost [41]. A k-nearest neighbor classifier labels a new observation by comparing it to the k-nearest training samples in a multi-dimensional space [42]. k = 5 was used for all experiments. ECOC is a classifier reduces a multiclass classification problem into a set of *L* binary classifiers. DendritePA uses a support vector machines (SVM) classifier for every pair of classes, *L* = 3. A new observation is assigned to the class that minimizes the losses of the *L* binary learners [43].

## Results

Our data set for segmentation consists of seven live fluorescence videos, which contain 3428 spines across all analyzed frames. TdTomato was used to label the entire structure of the cell (dendritic structural information) and wild type (wt)-Cofilin-GFP was used to label cofilin (cofilin distribution information). Videos varied in length from 39 frames and were collected at intervals varying from every 30 seconds to 60 seconds over approximately 20 minutes. All videos were collected at 40x magnification and were 128 by 128 in image resolution. To improve image quality, all frames were resized to 512 by 512 with bicubic interpolation [44]. Most of the segmentation algorithm parameters were kept constant for each video, except a multiplier for Otsu’s computed threshold which ranged from 0.2 to 0.4.

Dendrite segmentation was validated using the first frame of each of the seven videos. An expert in the field labeled each frame by selecting only regions that are a part of the dendrite and not the spines. Tests were performed using a GVF generated backbone, 2dSpAn’s convolution algorithm with piecewise linear approximation, and DendritePA after using a low pass filter. Results are obtained on a pixel detection basis and they are shown in Table 1. While DendritePA has a slightly worse precision, it has a much better recall.

**Table 1:** Dendrite segmentation results.

Method	Precision	Recall	True Positive	False Positive	False Negative
GVF Dendrite	75.22%	4.21%	4.21%	1.39%	95.79%
Piecewise 2dSpAn	75.97%	84.33%	84.33%	26.66%	15.67%
DendritePA	73.23%	90.32%	90.33%	33.01%	9.67%

Segmentation results for dendrites using GVF, 2dSpAn, and DendritePA.

**Fig 7:**
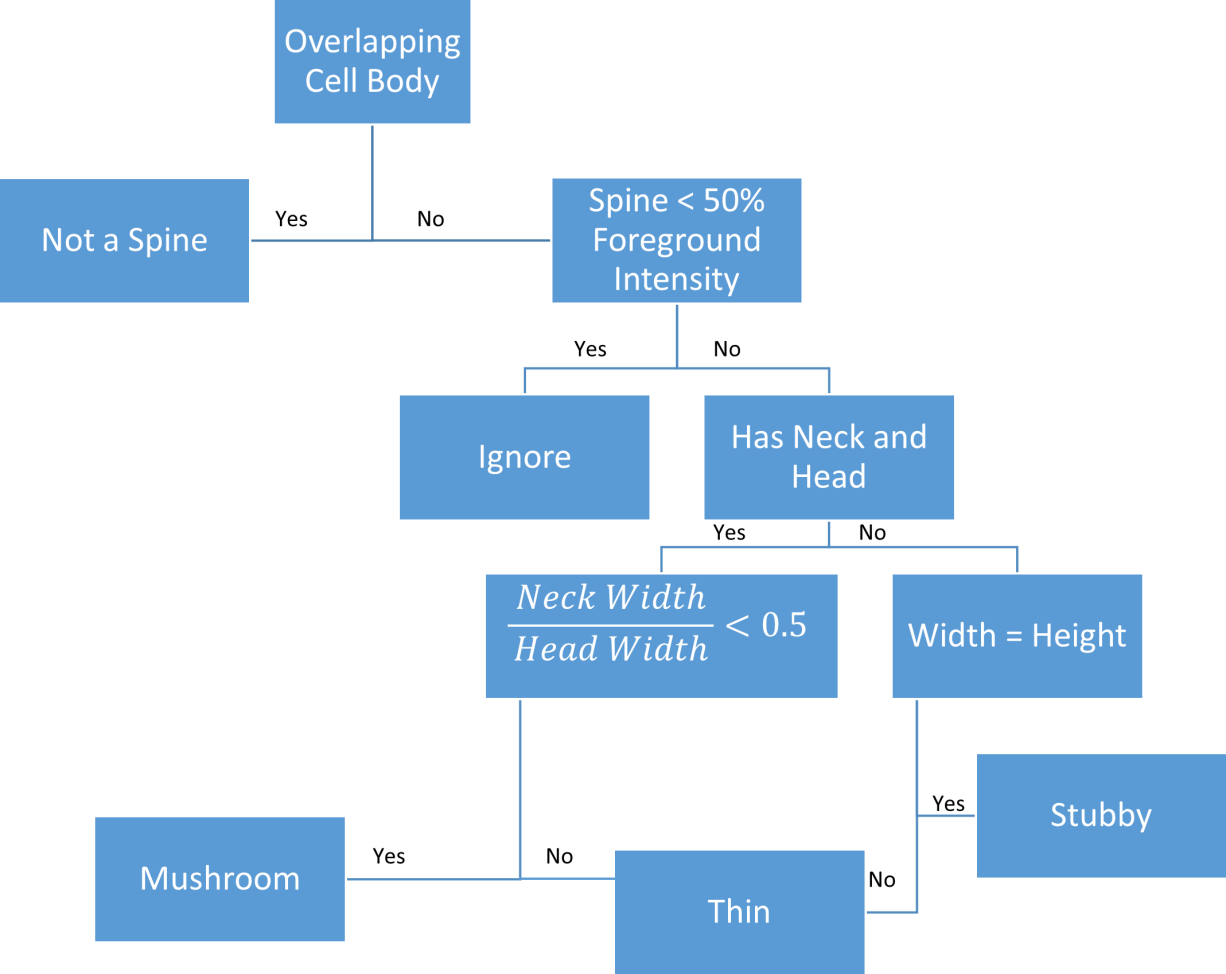
Ground-truth decision tree. Decision tree for generating ground truth of dendritic spine segmentation and classification. Spines in images were manually labelled (to be used as ground truth), using this decision tree to reduce bias and improve reproducibility. Spines were marked if they satisfied the conditions of the stubby, thin, or mushroom shapes.

Ground-truth for dendritic spine was created by labeling every spine in every frame manually by two experts in the field. In order to reduce user bias, the decision tree shown in Fig 7 was used as a guideline during ground-truth labeling. For comparison, our spine detection method is tested against the NeuronIQ software [45]. NeuronIQ was chosen because it is a fully automated dendritic spine segmentation software. 2dSpAn and NeuronStudio both required series of manual inputs for each frame, making them inappropriate for comparisons. While NeuronIQ was able to segment without preprocessing, using a TMIP to preprocess the videos improved segmentation results. A spine is considered detected if the segmentation has at least 50% overlap with the ground-truth data. Table 2 shows the results of segmentation for DendritePA, NeuronIQ with TMIP preprocessing, and NeuronIQ alone. NeuronIQ alone seems to produce many false positives. By using TMIP to preprocess the videos, the SNR is improved allowing NeuronIQ to perform better. DendritePA was able to achieve comparable recall, but could greatly improve precision as it has nearly half as many false positives. Because the classification of spines is dependent on the quality of their segmentations, only true positives were passed to the tracking and classification steps.

**Table 2:** Spine segmentation results.

Method	Precision	Recall	True Positive	False Positive	False Negative
NeuronIQ	28.13%	61.26%	61.26%	156.45%	38.74%
NeuronIQ with TMIP	53.69%	71.17%	71.18%	61.38%	28.82%
DendritePA	60.28%	61.93%	61.93%	40.64%	38.07%
DendritePA with TMIP	63.48%	67.24%	67.24%	38.68%	32.76%

Segmentation results for DendritePA, NeuronIQ with TMIP, and NeuronIQ alone.

DendritePA was also run without bicubic interpolation and compared to ground-truth sub-sampled at 128 by 128 resolution. Using the original resolution (128 by 128), DendritePA with TMIP had a precision of 59.00% and recall of 16.09%. These results are noticeably worse when compared to DendritePA with bicubic interpolation. To show that bicubic interpolation does not have a major effect on cofilin analysis, we computed the average intensity of cofilin in dendritic spines (green channel) for both 128 by 128 and 512 by 512. The average values for seven wild-type videos were 0.78 ± 0.13 for the original resolution and 0.76 ± 0.13 for the bicubic interpolation. The values were very similar between the two scales of resolution, and the standard deviation was identical. Also, because we are only interested in relative change of intensity compared to the previous frame, we do not require exact intensity values if the relationship between frames is maintained.

**Fig 8:**
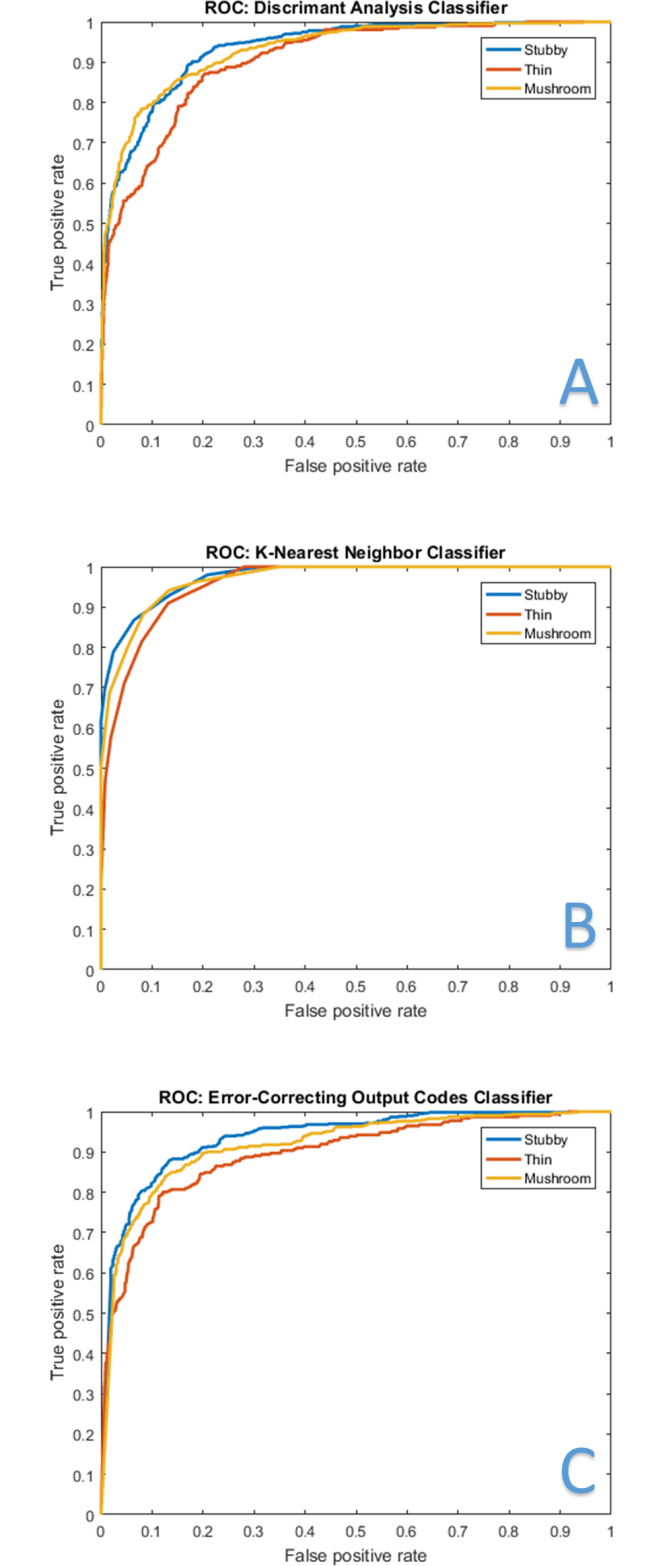
ROC plots. Receiver operating characteristic (ROC) curves for stubby (blue curves), thin (red curves), and mushroom (yellow curves) shaped spines. The ROC plots illustrate the ability of each classifier to distinguish the specific class when varying a discrimination threshold. A larger area under the curves (AUC) represents a better classifier. A) Discriminant analysis (DA) classifier. B) K-nearest neighbor (KNN) classifier. C) Error-correcting output codes (ECOC) classifier. K-nearest neighbor classifier had the largest AUC, proving to be the most accurate.

Classification of spines was done with 10-fold cross validation by partitioning the data into 10 equal sets. Each set is used as the test set once while the other 9 are used for training. The experiments were repeated 20 times by randomly shuffling the dataset so that the sets are randomly generated. Each spine was classified as stubby, thin or mushroom. While DendritePA is able to classify other phenotypes such as branched dendritic spines, it requires a training library with sufficient examples of the class. Because our data did not have enough branched spines, we focused our study on the three most common phenotypes [12, 46, 47]. Results are compared to a decision tree method that uses spine height and width features. Table 3 displays the classification results. The three classifiers using the proposed features outperform the traditional decision tree method by over 20%. The decision tree method was sensitive to small measurement errors at this resolution, while our proposed method is robust due to the spatiotemporal information in the spine energy images. While the classification rate of the classifiers performs well with all videos, a subset of videos was used to build the classifier used in later sections. Table 4 displays a confusion matrix of the three classifiers for videos 1–7 and Fig 8 shows the receiver operating characteristic curve (ROC) of the three classifiers for the same videos. ROC curves are useful in assessing the performance of an algorithm’s ability to detect an object. The larger the area under the curve (AUC), the better the classifier is at classifying the specific spine shape. From the Table 3 and the ROC plots, KNN slightly out performs the ECOC and DA classifiers.

**Table 3:** Classification results.

Classifier Results	DA	KNN	ECOC	Tree
**Video 1**	75.71 ± 0.47	81.35 ± 0.72	79.79 ± 0.90	52.19 ± 0.00
**Video 2**	59.90 ± 2.94	59.4 ± 3.32	54.10 ± 3.14	50.00 ± 0.00
**Video 3**	77.22 ± 0.82	75.36 ± 0.80	76.93 ± 0.89	63.79 ± 0.00
**Video 4**	81.97 ± 0.74	84.05 ± 0.69	81.33 ± 0.80	55.81 ± 0.00
**Video 5**	93.00 ± 0.72	98.26 ± 0.00	98.26 ± 0.00	79.52 ± 0.00
**Video 6**	76.34 ± 0.80	75.84 ± 0.75	74.55 ± 0.47	69.18 ± 0.00
**Video 7**	83.68 ± 1.07	90.44 ± 0.69	84.03 ± 0.43	58.49 ± 0.00
**Videos 1–7**	76.40 ± 0.29	80.61 ± 0.41	76.79 ± 0.33	62.10 ± 0.00

Classification results on ROIs 1–7 using DA, KNN, ECOC, and manual decision tree.

**Table 4:** Confusion matrices.

**Real Class**	**Predicted Class**			
	DA Classifier	***Stubby***	***Thin***	***Mushroom***
	***Stubby***	499	49	50
	***Thin***	62	192	57
	***Mushroom***	73	52	507
	KNN Classifier	***Stubby***	***Thin***	***Mushroom***
	***Stubby***	508	40	50
	***Thin***	62	192	57
	***Mushroom***	55	44	533
	ECOC Classifier	***Stubby***	***Thin***	***Mushroom***
	***Stubby***	526	27	45
	***Thin***	84	174	53
	***Mushroom***	98	50	484

Confusion Matrices for discriminant analysis, k-nearest neighbor, and error-correcting output codes.

**Fig 9:**
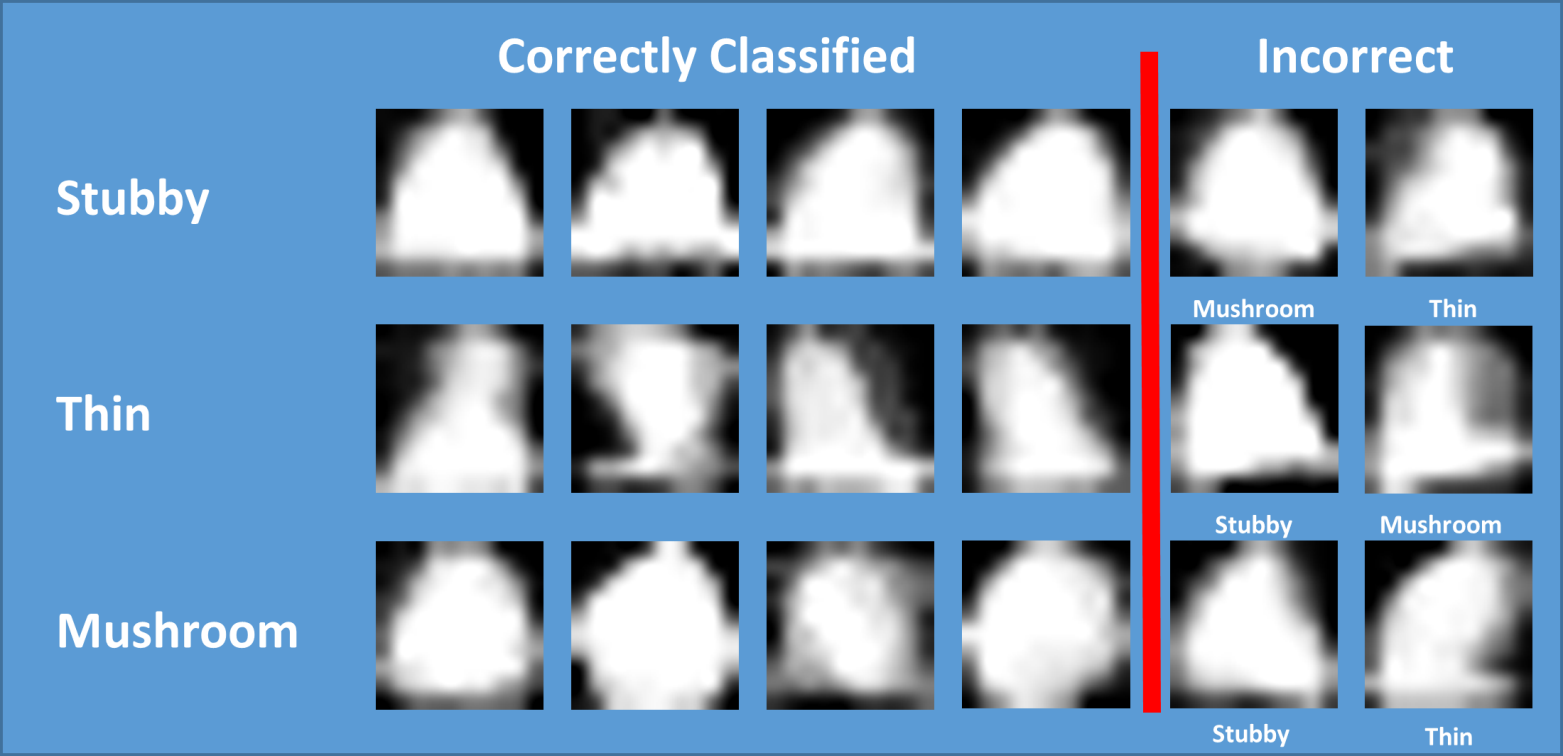
SEI classification examples. Each row displays spine energy images (SEI) that were classified by DendritePA as stubby, mushroom, or thin. The last two columns represent misclassified SEI, where the actual class of the spine track is shown below each SEI.

Upon closer examination of the SEI as shown in Fig 9, the SEI can be sorted visually into their mode spine class. Stubby spines tend to have a flat uniform base and a sharp apex. Thins have smaller middle regions and are blurry do to swaying over time. Mushrooms are round and have smaller bases. Fig 9 also displays SEIs that were misclassified. SEI seem to be commonly misclassified as stubby if they are triangular, as thin if they blurry or have an unusual base, and as mushroom if they are more circular.

### Cofilin conditions and spine shape

Three videos of S3A and three videos of S3D were collected using florescence microscopy. Each video has 40 frames and were captured at 40x magnification with 128 by 128 pixels. Like the wild-type videos, all S3A and S3D frames were resized to 512 by 512 using bicubic interpolation. TdTomato was used to label the actin cytoskeleton that make up the structure of the spine and CofilinS3A or CofilinS3D was used to label cofilin proteins. Visual observations of S3A show a relatively even distribution of stubby, thin and mushroom shaped spines. The density of spines were uneven across the video with some regions very dense and others less dense. For S3D, most spines were mushroom or stubby and had mild spine density.

**Fig 10:**
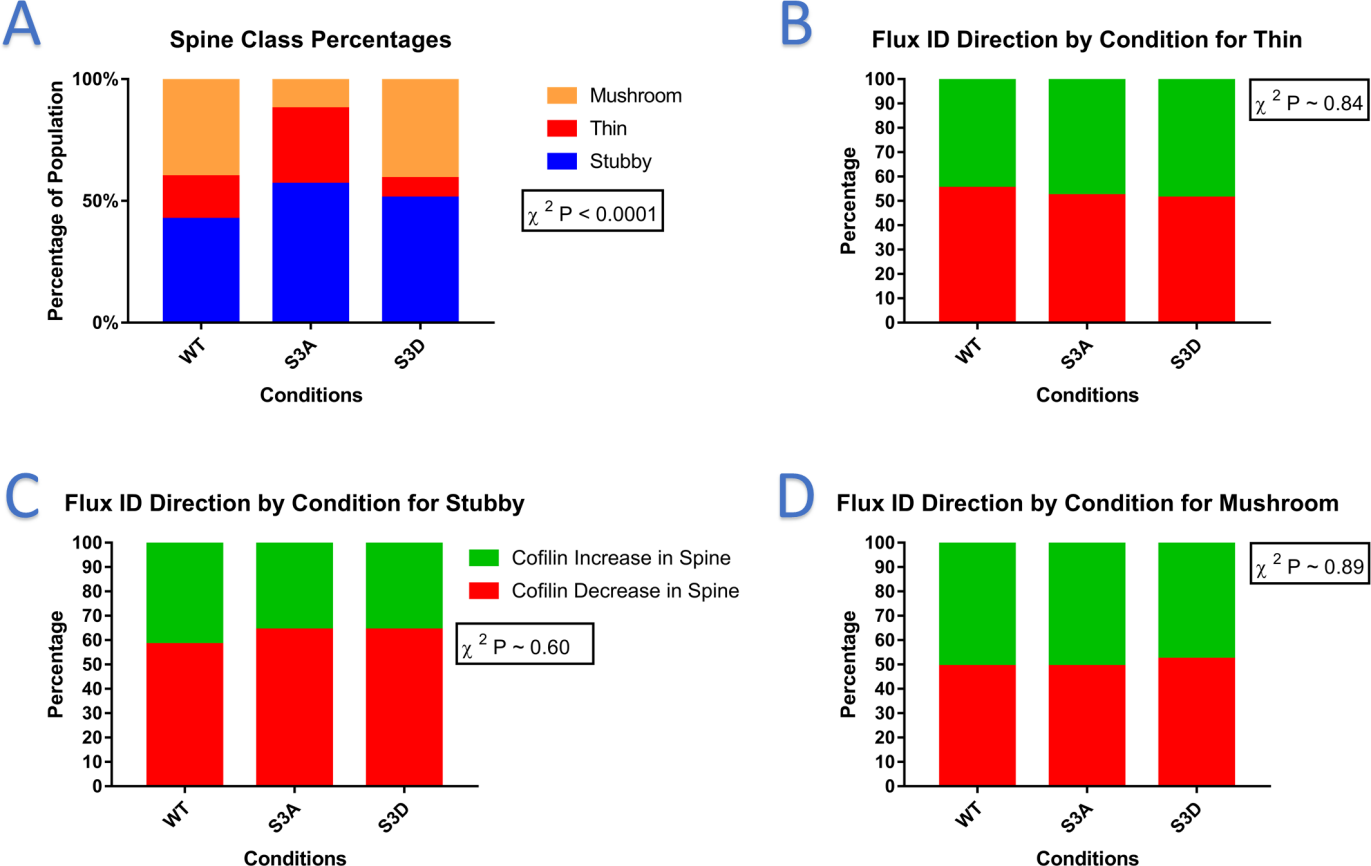
Cofilin-spine graphs. A) Bar graph of the percentage of detected spines classified as mushroom (orange), thin (red), stubby (blue) for each condition: WT, S3A, and S3D. The percentages for each condition were significantly different (P<0.0001 using Chi-squared test). B-D) Bar graphs of the flux direction computed with integrated density for B) Thin, C) stubby, and D) mushroom spines. For all cases, the percentages were not significantly different using Chi-squared analysis (thin p-value = 0.84, stubby p-value = 0.60, mushroom p-value = 0.89), indicating that cofilin flux has less importance on spine type.

DendritePA was used to segment and analyze videos showing the dendrite spines containing wt-cofilin, cofilinS3A, and cofilinS3D. A discriminant analysis classifier built with the ground truth was used to classify the data. Fig 10A shows the percentage of each spine class for the wild-type cofilin, cofilinS3A, and cofilinS3D videos. These percentages were significantly different (P < 0.0001 using Chi-squared test) and show that dendritic spine shape can be altered by introducing exogenous cofilin. For the case of the wild-type condition, most spines are stubby and mushroom, which indicate that spines are mostly mature for this condition. With the activation of cofilin in the cofilinS3A condition, there is a noticeable increase of thin spines and a decrease in mushrooms. This is consistent with the expectation that cofilin breaks down the F-actin in spines. With more cofilin being active the spines become less mature. Interestingly, we see that in the cofilinS3D videos there is a significant decrease in the number thin spines, while the ratio of stubby and mushroom spines remains the same. This indicates that by suppressing cofilin activation, it may be possible to induce more mature spines. Fig 10B–10D shows the percent of cofilin flux for each spine class across the three cofilin mutants. For all three spine classes, the ratio of cofilin flux moving in (cofilin density increases in spine, shown in green) or out of spines (cofilin density decreases in spine, shown in red) is consistent for all conditions and did not show significant difference (thin p-value = 0.84, stubby p-value = 0.60, mushroom p-value = 0.89). This indicates that the type of exogenous cofilin has little effect on the flow of cofilin. Also, because the ratio of in and out flux is constant within a spine class regardless of cofilin condition, cofilin flux has less importance in spine type than cofilin activation. In the time frame we examined (20 minutes), we did not notice a difference in the rate in which spine shape changed based on cofilin type. Although this was not our focus, it may be of interest to study in the future with longer videos.

Although endogenous cofilin is present in all three groups, the use of different exogenous cofilin is the only variable that is changed between the conditions. These exogenously expressed mutant forms of cofilin will affect spine morphology by competing with endogenous cofilin for binding to several actin proteins and enzymes. This is done to ensure that changes are due to only one alteration in the experimental setup. Because plasmid transfection efficiency is a concern, we measured the mean expression levels in all videos. The mean fluorescence intensity of cofilin S3A-GFP was 20.66 ± 1.20, cofilinS3D-GFP was 38.06 ± 12.54, and wt-cofilin was 23.29 ±7.94. The groups were shown to be not significantly different when compared using one-way ANOVA followed by Tukey’s multiple-comparison post-tests.

## Conclusions

In this paper, we have developed a pattern recognition software called DendritePA to analyze protein trafficking in neuronal florescence microscopy videos. Using spatiotemporal information, the DendritePA is able to enhance low contrast/low resolution images by computing a temporal maximum intensity projection which is used to improve the signal-to-noise ratio in every frame. Dendrite spines were automatically segmented using an improved kernel convolution method. Temporal dynamics of spines were used to generate a spine energy image, which is useful in classifying different spine shapes. Multiple classifiers were used to classify individual spine segmentations as stubby, thin, and mushroom. Lastly, we were able to estimate cofilin flux patterns and correlate them with the changing spine morphology over time. Mushroom/stubby spine shapes are recognized as mature/stable spines, whereas thin spines are classified as immature/unstable. By examining S3A and S3D conditions of cofilin, our data suggests that the level of activation of cofilin greatly affects the shape of spines. Highly activated cofilin seems to lead to structural instability of the spines. This is consistent with the actin-severing/remodeling function of cofilin.
